# Crowdsourcing-Based Indoor Semantic Map Construction and Localization Using Graph Optimization

**DOI:** 10.3390/s22166263

**Published:** 2022-08-20

**Authors:** Chao Li, Wennan Chai, Xiaohui Yang, Qingdang Li

**Affiliations:** 1 College of Automation and Electronic Engineering, Qingdao University of Science and Technology, Qingdao 266061, China; 2 College of Sino-German Institute Science and Technology, Qingdao University of Science and Technology, Qingdao 266061, China; 3 Faculty of Electrical Engineering and Computer Science, University of Kassel, 34132 Kassel, Germany

**Keywords:** crowdsourcing, graph optimization, localization, mapping, multi-sensor fusion, object detector

## Abstract

The advancement of smartphones with multiple built-in sensors facilitates the development of crowdsourcing-based indoor map construction and localization. This paper proposes a crowdsourcing-based indoor semantic map construction and localization method using graph optimization. Using waypoints, semantic landmarks, and Wi-Fi landmarks as nodes and the relevance between waypoints and landmarks (i.e., waypoint–waypoint, waypoint–semantic, waypoint–Wi-Fi, semantic–semantic, and Wi-Fi–Wi-Fi) as edges, the optimization graph is constructed. Initializing the venue map is the single-track semantic map with the highest quality, as determined by a proposed map quality evaluation function. The aligned venue and candidate maps are optimized while satisfying the constraints, with the candidate map exhibiting the highest degree of similarity to the venue map. The lightweight venue map is then updated in terms of waypoint and landmark attributes, as well as the relationship between waypoints and landmarks. To determine a pedestrian’s location on a venue map, similarities between a local map and a venue map are evaluated. Experiments conducted in an office building and shopping mall scenes demonstrate that crowdsourcing-based venue maps are superior to single-track semantic maps. Additionally, the landmark matching-based localization method can achieve a mean localization error of less than 0.5 m on the venue map, compared to 0.6 m in a single-track semantic map.

## 1. Introduction

An indoor map is crucial for user-end localization [1], indoor navigation [2], and the drift constraint of inertial sensors [3,4]. However, because the geometric features and signal sources in indoor environments tend to change dynamically over time, traditional manual operation-based map construction methods encounter difficulties in updating. Additionally, digital or computer-aided design (CAD) maps describing environments are usually unavailable due to commercial interest or privacy [5,6]. Therefore, in both industry and academia, the autonomous construction and updating of a high-precision and robust venue map in unknown indoor environments has been a hot topic.

Scholars have proposed many sensor-based mapping solutions, such as lidar-based [7], camera-based [8], Wi-Fi-based, inertial measurement unit (IMU)-based, and magnetic-based [9], to solve the mapping problem in unknown indoor environments. Due to these sensors’ inherent characteristics, single-sensor-based map construction methods have limited application scopes. Multipath interference and packet loss, for instance, significantly impact the stability of Wi-Fi signals, which can result in the loss or misidentification of Wi-Fi fingerprints. Furthermore, it is time-consuming and labor-intensive to construct a radio map based on offline-collected Wi-Fi data at reference points (RPs) [10,11]. The construction of high-accuracy maps is feasible with multi-sensor fusion-based solutions that leverage sensor complementarity. However, a single-track map based on multi-sensor fusion contains limited space-related information about the entire scene. Three factors have a significant effect on the accuracy of a single-track map: (1) signal stability, (2) trajectory length, and (3) the stability of hardware devices (such as robots, smartphones, and unmanned aerial vehicles (UAVs)). Fusing multiple single-track maps is a feasible solution for constructing high-accuracy and wide-coverage venue maps, where the raw sensor data are collected by a new sensing paradigm, crowdsourcing [12]. Smartphones, which are portable smart terminals, have superior environmental perception thanks to an array of built-in sensors, including cameras [13], IMUs, Wi-Fi signal receivers, magnetometers [9], and photoelectric encoders. Due to the prevalence of smartphones, the efficiency of crowdsourcing data collection is also enhanced. In complex environments, numerous smartphone-based map construction and localization methods via crowdsourcing have been proposed [14], such as CrowdInside [15], SISE [16], and Zhou et al. also proposed methods [17,18,19]. By processing smartphone-collected crowdsourcing data, a map is constructed by combining semantic/feature information with the estimated trajectories. Trajectories are estimated using pedestrian dead reckoning (PDR) [18,20] or visual SLAM [21].

Landmarks play a crucial role in crowdsourcing-based or multi-robot cooperative mapping, which can be used for trajectory alignment and venue characterization [22]. Semantic landmarks, such as elevators, corners, stairs, and escalators, are detected by activity detection algorithms [17] based on inertial sensors or barometers. However, an indoor environment typically contains more than the predefined number of semantic landmarks, such as ashcans, windows, and doors. Theoretically, as the variety of landmarks available for object detection increases, so does the density of landmarks. Rich low- (such as colors, contexts, and points) and high-level visual features (e.g., semantics) are presented in an image. Moreover, due to the development of deep learning, increasingly precise object detectors [23,24] and semantic segmentation models [25,26] are being used for semantic landmark recognition. Landmarks can also be used as nodes of an optimization graph, such as [17,18,27]. Since the single-track semantic maps consist of waypoints, semantic landmarks, and Wi-Fi landmarks, we construct a novel optimization graph for map optimization instead of other classic optimization methods, such as particle swarm optimization [28], hybrid grey wolf optimizer [29], artificial bee colony optimization algorithm [30], and genetic algorithm [31].

This paper proposes a crowdsourcing-based semantic map construction and updating method for unknown indoor environments, such as office buildings, shopping malls, and disaster-stricken houses. The constructed map can be used as a localization map for estimating a client’s location. The proposed method combines the idea of PDR-aided VI-SLAM [32], object detection and crowdsourcing, and uses the optimization technique to fuse crowdsourced trajectories collected by different clients. Using images, inertial measurements, and Wi-Fi signals constructs a single-track semantic map composed of waypoints, semantic landmarks, and Wi-Fi landmarks. The highest-quality single-track map is used to initialize the venue map to ensure mapping precision and efficacy. A candidate map for map fusion is the single-track semantic map with the highest degree of similarity to the venue map. Due to the vacancy of the actual azimuth of starting points, trajectories estimated by PDR-aided visual-inertial simultaneous mapping and localization (PDR-aided VI-SLAM) are in an inconsistent coordinate system. Consequently, the candidate and venue maps are aligned using corresponding landmarks before employing graph optimization. The nodes of the optimization graph are waypoints, semantic landmarks, and Wi-Fi landmarks. And the edges are association constraints (such as waypoint–waypoint, waypoint–Wi-Fi, and waypoint–semantic) and matching constraints (such as semantic–semantic and Wi-Fi–Wi-Fi). The lightweight venue map is updated after graph optimization with respect to waypoints, semantic landmarks, and Wi-Fi landmarks. To estimate the location of a pedestrian in relation to the venue map, a local map is compared to the venue map. The proposed method has broad application prospects, such as indoor navigation in unknown environments, big data intelligent recommendation, and post-disaster search and rescue. Taking indoor navigation in unknown environments as an example, the proposed method solves the problem of map construction for unknown indoor environments and indoor positioning drift. By processing the data collected by different mall shoppers, a venue map describing the whole shopping mall is constructed without any prior knowledge. Using the proposed localization methods, a pedestrian’s location on the venue map is estimated. After that, possible pathways from the current position to the destination are accessible. The multi-landmark matching-based localization method improves the localization robustness significantly.

To summarize, the main contributions of the proposed method are:

- A crowdsourcing-based semantic map construction and updating method are proposed for unknown indoor environments, which can significantly reduce the cost of map construction and updating. Particularly, the crowdsourcing data is collected using smartphones’ built-in sensors.
- An optimization graph is constructed using waypoints, semantic landmarks, and Wi-Fi landmarks as nodes, and the relevance between waypoints and landmarks as edges, which improves the accuracy of venue maps.
- The real-world experimental results demonstrate that the proposed map construction and updating method is suitable for office building and shopping mall scenes. Additionally, venue maps have higher accuracy than single-track semantic maps when used for localization.

The remainder of this paper is organized as follows. Section 2 reviews the related works of the proposed method. Section 3 describes the main contexts in detail, while Section 3.1 provides an overview of the system. Section 4 presents an analysis of experiment results. Finally, Section 5 concludes the paper with a summary and a discussion of future research directions.

## 2. Related Works

### 2.1. Crowdsourcing-Based Map Construction

Crowdsourcing is a feasible method for improving mapping precision [33]. Crowdsourcing-based CrowdInside refined the internal features of a floorplan through trace segmentation, segment classification, and clustering. Additionally, it used the alpha shape to obtain the overall floorplan shape [15]. SISE proposed enGraph, a new abstraction data model for representing indoor entities and corresponding semantics [16]. In 2015, Zhou et al. [17] developed a link-node optimization model for indoor mapping, with pathways representing links and activity landmarks representing nodes. The activity landmarks detected by the activity detection algorithm were grouped into distinct node clusters based on their sequence and spatial characteristics. All the dispersed nodes were linked with straight lines. However, many detailed features between nodes were disregarded when using a direct connection. Humans, for instance, choose a path autonomously based on the actual environments, such as avoiding obstacles. Therefore, trajectories were more suitable than straight lines for describing a scene. Based on this, Du et al. constructed a crowdsourcing-based radio map by matching the PDR-estimated trajectories with the candidate routes based on their proposed shape context algorithm [20]. Zhou et al. [18] proposed a method for constructing indoor maps by coupling landmarks to PDR-estimated trajectories. Due to the redundancy of the trajectory, the alignment matrix was calculated in an incremental manner. To reduce the redundancy of a constructed map, trajectory segments with high similarities were fused using the Dynamic Time Warping (DTW) algorithm [19] to evaluate the similarities. The redundant data were removed by a coefficient weight algorithm together with the scoring matrix [34]. This paper used the PDR-aided VI-SLAM to estimate the mapping trajectories. At the same time, the redundant data was removed by map fusion and updating.

Before fusion or optimization, trajectories estimated using different user-ends’ crowdsourcing data must be aligned due to coordinate inconsistency. Likewise, multi-platform cooperative mapping also has a problem with coordinate initialization. Zhu et al. [22] proposed the attribute similarity principle, the topology similarity principle, and the iterative closest point (ICP) principle to evaluate the similarities between local maps to align multiple maps. Local maps constructed by fusing heterogeneous sensor data collected by multiple robots were shared to construct global scene maps. Yue et al. [35] proposed a probabilistic map matching (PMM) algorithm for structural and voxel features. Pre-matching based on structural features improved the accuracy and efficiency of voxel feature-based matching. Additionally, they proposed an expectation-maximization approach for data association between local maps [36]. In addition to calculating the geometry occupancy probability, stitching the overlapping areas was a problem-solving concept [37], such as ICP for point cloud and landmark matching [38]. Clustering was also a practicable solution for map alignment. Shu et al. [39] proposed a trajectory segmentation and clustering algorithm based on improved discrete Fréchet distance and entropy theory.

Landmark-based indoor map construction methods are predicated on the following assumptions: (1) the number of landmarks exceeds the predetermined threshold, and (2) landmarks can be detected by multiple trajectories [27]. As a result of the availability of numerous trajectories through crowdsourcing, the landmarks detected by activity detection algorithms are sparse and dispersed, which may result in map fusion or matching failures. The object detector or semantic segmentation methods based on deep learning have excellent detection accuracy and efficiency. Consequently, this paper employs the YOLO V3 detector to identify semantic objects in the selected keyframes. Wi-Fi landmarks are also extracted to increase the density of landmarks, and they can also be utilized for fingerprinting- and landmark-matching-based localization.

### 2.2. Graph Optimization-Based Map Construction

Many scholars have used graph optimization to solve the problem of map construction. The core of graph optimization was the construction of a graph, namely, an error energy function. To construct a Wi-Fi radio map for the site survey phase, Tan et al. [40] used PDR- and Wi-Fi-based edges to represent position constraints between two raw poses and landmark-based edges to represent constraints for a single pose. Zuo et al. [41] utilized a PDR algorithm to estimate the distance constraint between adjacent poses, a BLE fingerprinting method to constrain poses with similar fingerprints, and a path-loss model to constrain the distance between the poses and the beacons. The Global Navigation Satellite System (GNSS) provided a large amount of high-accurate position information for multi-platform clients in outdoor environments. GVINS was a state estimator using GNSS raw measurements, inertial measurements, and visual images. Its constraint factors included inertial factors, visual factors, code pseud-orange and Doppler factors [42]. Similarly, Das et al. modeled multiple optimization graphs using visual information from a precise stereo camera-based visual odometry, inertial information from a vehicle velocity and yaw-rate sensor-based odometry, and GNSS information [43]. The FGO-NDT method reduced the drift errors of systems by using a factor graph, which combined the GNSS location and loop information [44]. GraphIPS constructed an optimization graph with location nodes (LNs) and sensing nodes (SNs). LNs and SNs were constrained by LN-SN distance, adjacent step distance, and nonadjacent step distance, which were calculated using received signal strength (RSS), accelerometer, and angle-of-arrival (AoA) data, respectively [45]. Zhou et al. [18] proposed a two-step method for indoor map optimization. The first-step optimization graph was constructed using transformation matrices as nodes and the errors of the transformed results as edges. The second-step optimization was the pose global optimization (PGO), which consisted of inner and outer constraints. The former denoted the position relationship between neighbor poses, whereas the latter denoted the intersected loop position poses (LPP) of different trajectories. The 80% error range for the two-step optimization-based method in an application scene was about 1.7–3.5 m. A graph could also be used for indoor Wi-Fi radio map abstraction, where the activity landmarks were employed as nodes, and the possible user path was employed as edges [38].

By fusing crowdsourced data into a graph-based formulation, it is possible to significantly improve the map’s accuracy [38]. Therefore, this paper proposes a graph optimization-based method for indoor map construction and localization. To reduce the requirement for computing power, the highest-quality single-track map is used to initialize the venue map. Using graph optimization, the single-track map with the highest degree of matching to the venue map is also fused. The venue map is updated after optimization in terms of waypoints, semantic landmarks, and Wi-Fi landmarks.

## 3. The Main Context

### 3.1. System Overview

**Figure 1:**
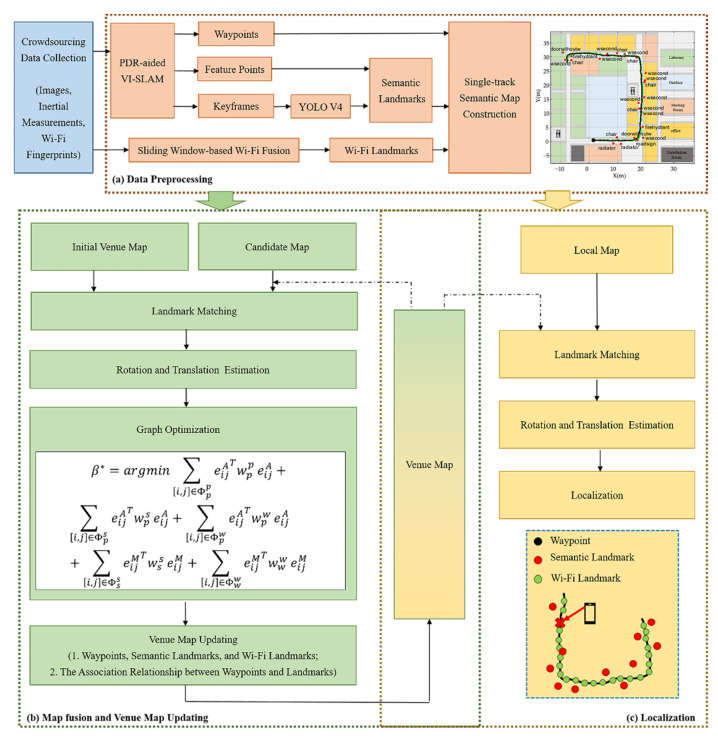
The system overview.

The map construction and localization method are depicted in Figure 1 with a general overview. It relies on smartphone-collected sensor data, including visual images, inertial measurements (i.e., accelerometer and gyroscope measurements), and raw Wi-Fi fingerprints. Specifically, Wi-Fi fingerprints contain the media address control (MAC) and RSS values of access points (APs).

Data preprocessing is the first step of the crowdsourcing-based indoor semantic map construction and localization method. After fusing multi-sensor data, a group of single-track semantic maps comprised of waypoints, semantic landmarks, and Wi-Fi landmarks is constructed.

The second component involves map fusion and venue map updates. First, the transformation matrix between the candidate and venue maps is estimated using semantic and Wi-Fi landmarks that match. Then, optimize the aligned maps under the constraints of association and matching. The waypoints, semantic landmarks, and Wi-Fi landmarks serve as nodes in the optimization graph, while the association and matching relationships between waypoints and landmarks serve as constraint edges. To reduce venue map redundancy, the optimized venue map is updated with respect to waypoints, semantic landmarks, Wi-Fi landmarks, and the association between them. Notably, map alignment and fusion are performed incrementally. When a new candidate map is selected, a new map alignment and venue map update iteration is initiated.

The final component is localization. A localization method based on landmark matching is utilized to estimate a pedestrian’s location on a venue map. Wi-Fi fingerprinting specifically determines the relationship between semantic landmarks in the local and venue maps.

Section 3.2, Section 3.3, Section 3.4, Section 3.5 and Section 3.6 contain more detail. The proposed optimization graph is a critical insight.

### 3.2. Crowdsourcing Data Preprocessing

Smartphone-collected crowdsourcing data is preprocessed to construct single-track semantic maps containing waypoints, semantic landmarks, and Wi-Fi landmarks.

The PDR-aided VI-SLAM outputs keyframes, keyframe-rate feature points, and IMU-rate waypoints using time-synchronized monocular visual and inertial measurements as inputs. The PDR-aided VI-SLAM uses the PDR’s velocity as an external observation to constrain the scale drift of the conventional VI-SLAM systems [32], which is defined as Equation (1):

(1) $$v_{t}^{PAM}=\lambda_{PDR}v_{t}^{PDR}+\lambda_{VIO}v_{t}^{VIO}$$

where $v_{t}^{PAM}$, $v_{t}^{PDR}$, and $v_{t}^{VIO}$ denote a pedestrian’s velocity at time t, which are estimated by the PDR-aided VI-SLAM (abbreviated, PAM), PDR, and visual and inertial odometry (abbreviated, VIO), respectively. $\lambda_{PDR}$ and $\lambda_{VIO}$ denote the weight factor of the velocity estimated by the PDR and VIO, respectively. Visual tracking may fail due to a change in lighting or a lack of texture, where PDR has the greatest weight in velocity estimation, i.e., $\lambda_{VIO}=1$.

The PDR-aided VI-SLAM is applicable to closed-loop and non-closed-loop trajectory scale correction. Experiments conducted on the self-collected and public ADVIO datasets [32,46,47] confirmed that PDR-aided VI-SLAM provides more accurate pose estimation than traditional VI-SLAM systems [32]. The front-end visual processing outputs keyframes based on the average parallax and tracking quality principles [48]. When YOLO V4 only detects semantic objects in the selected keyframes, the object detector’s performance is significantly enhanced.

As summarized in Table 1, YOLO V4, a pretrained object detector, outputs the attributes of semantic objects in the selected keyframes with high precision. Additionally, it summarizes the attributes of the PDR-aided VI-SLAM-estimated feature points. By analyzing their attributes, it is possible to conclude that a semantic landmark can be created using the shared attribute of semantic objects and feature points, namely, 2D pixel coordinates.

For the (g)th keyframe, feature points satisfying the constraint of Equation (2) correspond to the (l)th semantic object:

(2) $$\begin{array}{c} x_{min}^{g,l}\leq u_{k}^{fp}\leq x_{max}^{g,l}\\ y_{min}^{g,l}\leq v_{k}^{fp}\leq y_{max}^{g,l} \end{array}$$

where $\left(x_{min},y_{min}\right)$ is the upper left vertex of the semantic bounding box, and $\left(x_{max},y_{max}\right)$ is the lower right vertex. (u,v) is the pixel coordinate of a feature point.

The intersection over union (IOU) metric evaluates the coincidence degree of the (l)th semantic object with those objects having the same class label in previously n keyframes. If the IOU score of the (l)th semantic object exceeds the set threshold, the semantic object and corresponding feature points are labeled with an index, which is the same as that of the matched semantic objects in previously n keyframes. Since the bounding box is rectangular and semantic objects are typical of irregular shapes, the bounding box contains outliers. The R-DBSCAN algorithm is applied to filter outliers from randomly selected feature points. The final step involves calculating the location of a semantic object relative to the corresponding trajectory as the centroid of filtered feature points with the same index.

Wi-Fi fingerprint stability is significantly impacted by the signal multipath effect, air humidity, and access channel occupancy [34]. Therefore, we employ a sliding window-based Wi-Fi fusion algorithm to improve the stability of the AP. In a sliding window, APs collected more than once are defined as shared APs, while others are defined as unique APs. The shared and unique APs in a sliding window form a Wi-Fi landmark, with the shared APs, fused in terms of RSS values and maturity. RSS values are used to sort all the APs belonging to a Wi-Fi landmark. The sliding window-based Wi-Fi fusion algorithm is an offline and efficient Wi-Fi fingerprint construction method as compared to the manual-based method. The Wi-Fi landmark can also be used as a localization feature.

**Figure 2:**
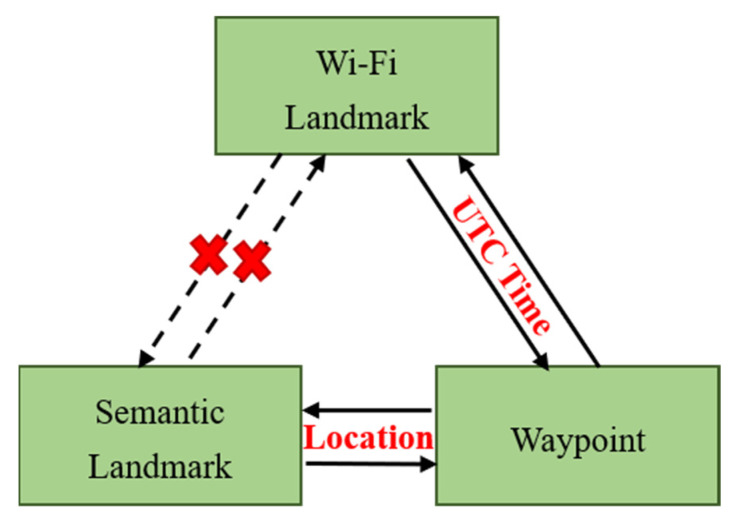
The association relationship among the waypoints, semantic landmarks, and Wi-Fi landmarks.

A single-track semantic map is a map that depicts the environment’s spatial characteristics. Figure 2 depicts the association relationship between waypoints, semantic landmarks, and Wi-Fi landmarks, where Wi-Fi landmarks have no direct association relationship with semantic landmarks. As a result, waypoints are employed as a link between semantic and Wi-Fi landmarks. Only waypoints, semantic landmarks, and Wi-Fi landmarks are saved in a map file to reduce storage requirements.

### 3.3. Map Alignment

All single-track semantic maps are constructed following the crowdsourcing data preprocessing. To avoid the effect of low-quality single-track semantic maps on map fusion, the quality of a map is evaluated using the function shown in Equation (3). Low-quality single-track semantic maps are filtered out, and the map with the highest quality score is chosen as the initial venue map. In Equation (3), a map quality $Score_{Map}$ is positively correlated with the landmarks’ quality on a map. For conciseness, the abbreviated alphabets in the following equations are defined as follows: the waypoints are denoted as “P”, the semantic landmarks are denoted as “S”, the Wi-Fi landmarks are denoted as “W”, the association relationship is denoted as “A”, the matching relationship is denoted as “M”, the venue map is denoted as “V”, the candidate map is denoted as “C”, and the local map is denoted as “L”.

(3) $$Score_{Map}=\mu_{S}\times N_{S}\times \bar{conf}+\mu_{W}\times N_{W}$$

where $\mu_{S}$ and $\mu_{W}$ denote the importance of the semantic and Wi-Fi landmarks in evaluating the map quality, respectively. Compared to environmentally sensitive Wi-Fi landmarks, semantic landmarks are more stable and reliable. Therefore, $\mu_{S}$ is twice as large as $\mu_{W}$. We also take the number of semantic landmarks $N_{S}$ and Wi-Fi landmarks $N_{w}$ into consideration. The map’s confidence $\bar{conf}$ is defined as:

(4) $$\bar{conf}=\frac{1}{N_{S}}\overset{N_{S}}{\underset{g=1}{\sum }}conf_{g}$$

For the (h)th semantic landmark of a single-track semantic map, the confidence value $conf_{h}$ reflects how confident the detector is that the bounding box contains an object and how accurate it thinks the bounding box is that it predicts.

**Figure 3:**
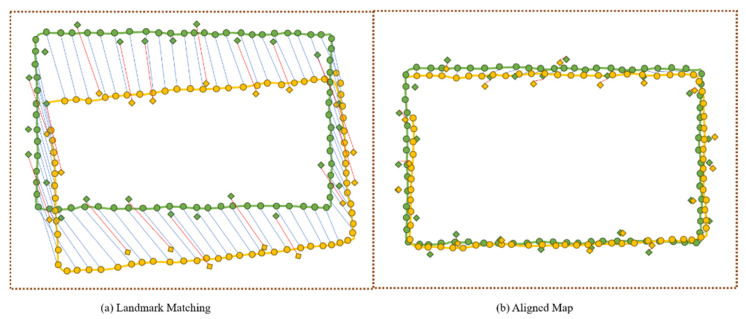
The schematic diagram of map alignment. (**a**) The process of landmark matching, and (**b**) the aligned maps after transformation.

Figure 3 illustrates the schematic diagram of map alignment. For map alignment, the semantic landmarks (diamonds) and Wi-Fi landmarks (circles) on a candidate map (colored green) are matched with those on a venue map (colored yellow). Map alignment is performed in an incremental method; when a candidate map is chosen, a standalone map alignment and venue map update are generated in one iteration.

The matching quality of venue and candidate maps $Score_{M}$ relies on the matched Wi-Fi and semantic landmarks, as defined in Equation (5):

(5) $$Score_{M}={\left(\eta_{1}\times Dis_{W-W}+\eta_{2}\times Dis_{S-S}\right)}^{-1}$$

The weight factor of the Wi-Fi landmarks $\eta_{1}$ and semantic landmarks $\eta_{2}$ are preset for evaluating the map matching quality. Considering the (m)th Wi-Fi fingerprint in a venue map has n APs, the RSS values of the n APs are $\left(RSS_{1}{}^{m},RSS_{2}{}^{m},\ldots ,RSS_{n-1}{}^{m},RSS_{n}{}^{m}\right)$, and considering the (r)th Wi-Fi fingerprint in the single-track semantic map to be matched has s APs, the RSS values of the s APs are $\left(RSS_{1}{}^{r},RSS_{2}{}^{r},\ldots ,RSS_{s-1}{}^{r},RSS_{s}{}^{r}\right)$. Suppose there are k shared APs, k≤s≤n, the Wi-Fi Euclidean distance $Euc_{W}$ is calculated as follows:

(6) $$Euc_{W}=\sqrt{\overset{k}{\underset{o=1}{\sum }}{\left(RSS_{o}{}^{m}-RSS_{o}{}^{r}\right)}^{2}+\overset{n}{\underset{p=n-k}{\sum }}{\left(RSS_{p}{}^{m}-RSS_{\varepsilon }\right)}^{2}+\overset{s}{\underset{q=s-k}{\sum }}{\left(RSS_{q}{}^{r}-RSS_{\varepsilon }\right)}^{2}}$$

For the (n−k) unique APs in the (m)th Wi-Fi fingerprint, and the (s−k) unique APs in the (r)th Wi-Fi fingerprint, the RSS value is unknown. Therefore, to unify RSS sequence length, the missing RSS values $RSS_{\varepsilon }$ of unique APs in the (m)th, and (r)th Wi-Fi fingerprints are set as −99.

To reduce the effect of moving APs on the Wi-Fi landmark matching, the Wi-Fi sequence distance $Seq_{W}$ is calculated as [49]:

(7) $$Seq_{W}=\|Seq^{m}-Seq^{r}{\|}_{2}$$

The Wi-Fi landmark distance between the venue map and single-track semantic map to be matched $Dis_{W}$ is determined by the Wi-Fi Euclidean distance $Euc_{W}$ and Wi-Fi sequence distance $Seq_{W}$ simultaneously, as defined in Equation (7):

(8) $$Dis_{W}=\xi_{1}\times Euc_{W}+\xi_{2}\times Seq_{W}$$

The moving APs can be detected by an AP selection algorithm [50]. Therefore, the weight factor of Wi-Fi Euclidean distance $\xi_{1}$ and Wi-Fi sequence distance $\xi_{2}$ can be dynamically adjusted based on the corresponding AP quality. Additionally, the matching score can be used as prior knowledge for the subsequent iteration of selecting a candidate map.

The semantic landmarks are pre-matched based on their class attributes, establishing a one-to-many association. Since semantic landmarks are associated with Wi-Fi landmarks, Wi-Fi fingerprinting is used to determine a one-to-one association between semantic landmarks. Additionally, the distance between semantic landmarks is calculated using Equations (6)–(8). Utilizing pre-matching significantly improves the efficiency of matching landmarks.

As shown in Figure 3a, a topological matching relationship is established between the venue and candidate maps using the matched landmark pairs (i.e., Wi-Fi and semantic landmark pairs). To align the candidate and venue maps, a transformation matrix $T_{V}^{C}$ is estimated by minimizing the Equation (9):

(9) $$\underset{V}{\sum }w_{V}\times {\left(L_{V}^{S}-T_{V}^{C}L_{C}^{S}\right)}^{2}+\underset{C}{\sum }w_{C}\times {\left(L_{V}^{W}-T_{V}^{C}L_{C}^{W}\right)}^{2}$$

where $L_{V}^{S}$ and $L_{V}^{W}$ denote the matched semantic and Wi-Fi landmarks in a venue map, respectively; $L_{C}^{S}$ and $L_{C}^{W}$ are the matched semantic and Wi-Fi landmarks in a candidate map, respectively; The weight factor of the matched semantic landmarks $w_{V}$ is higher than that of Wi-Fi landmarks $w_{C}$ in transformation matrix estimation, which is consistent with the map quality evaluation and map matching score calculation.

### 3.4. Graph Optimization

**Figure 4:**
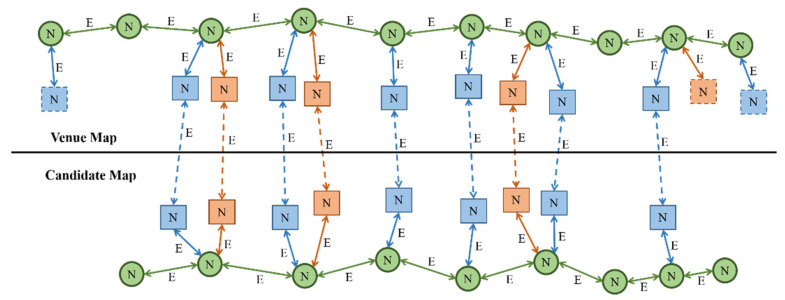
The schematic diagram of the optimization graph.

Graph optimization aims to improve the maximum fit of aligned maps by optimizing the position of waypoints and associated landmarks while satisfying association and matching constraints. Figure 4 elaborates on the relationship between optimization graph nodes and edges. The association constraints (solid lines) in the optimization graph are the edges connecting waypoints and waypoints (solid green lines), waypoints and semantic landmarks (solid red lines), and waypoints and Wi-Fi landmarks (solid blue lines). Compared to the association constraint within one single map, the matching constraint edges (dotted lines) connect the matched landmarks between the venue and candidate maps, including semantic–semantic (dotted red lines) and Wi-Fi–Wi-Fi (dotted blue lines). Nodes are the “N”-marked groups of waypoints, semantic landmarks, and Wi-Fi landmarks observed on maps. All the edges are marked with “E”. There are also unmatched landmarks on the candidate and venue maps, as indicated by the dotted boxes.

For the (i)th and (j)th landmarks in a venue or candidate map, their absolute poses $T_{i}$ and $T_{j}$ are denoted by Equations (10) and (11), respectively [18]:

(10) $$T_{i}=\left[\begin{array}{cc} R_{i} & t_{i}\\ 0 & 1 \end{array}\right]$$

(11) $$T_{j}=\left[\begin{array}{cc} R_{j} & t_{j}\\ 0 & 1 \end{array}\right]$$

where $R_{i}$ and $R_{j}$ denote the rotation matrix of the (i)th and (j)th landmarks relative to the initial pose point, respectively. $t_{i}$ and $t_{j}$ denote the translation vector of the (i)th and (j)th landmarks relative to the initial pose point, respectively.

Therefore, the pose of the (j)th landmark relative to the (i)th landmark $T_{ij}$ is calculated as follows:

(12) $$T_{ij}=T_{i}^{-1}T_{j}=\left[\begin{array}{cc} R_{i}^{T}R_{j} & R_{i}^{T}t_{j}-R_{i}^{T}t_{i}\\ 0 & 1 \end{array}\right]=\left[\begin{array}{cc} R\left(\phi_{j}-\phi_{i}\right) & R^{T}\left(\phi_{i}\right)\left(t_{j}-t_{i}\right)\\ 0 & 1 \end{array}\right]$$

where $\phi_{i}$ and $\phi_{j}$ are the angle of the (i)th and (j)th landmark relative to the initial pose point, respectively.

There are two types of relationships between the (i)th and (j)th landmarks: the association relationship and the matching relationship. If the (i)th landmark is associated with the (j)th landmark, relative pose measurement ${\tilde{T}}_{ij}$ is:

(13) $${\tilde{T}}_{ij}=\left[\begin{array}{cc} R_{ij}\left({\tilde{\phi }}_{ij}\right) & {\tilde{t}}_{ij}\\ 0 & 1 \end{array}\right]$$

Then, the pose error is:

(14) $${\tilde{T}}_{ij}^{-1}T_{ij}=\left[\begin{array}{cc} R^{T}\left({\tilde{\phi }}_{ij}\right)R\left(\phi_{j}-\phi_{i}\right) & R^{T}\left({\tilde{\phi }}_{ij}\right)R^{T}\left(\phi_{i}\right)\left(t_{j}-t_{i}\right)-R^{T}\left({\tilde{\phi }}_{ij}\right){\tilde{t}}_{ij}\\ 0 & 1 \end{array}\right]$$

The cost function in terms of position and angle for the associated landmarks $e_{ij}^{A}$ is defined as:

(15) $$e_{ij}^{A}=\left[\begin{array}{c} R^{T}\left({\tilde{\phi }}_{ij}\right)R^{T}\left(\phi_{i}\right)\left(t_{j}-t_{i}\right)-R^{T}\left({\tilde{\phi }}_{ij}\right){\tilde{t}}_{ij}\\ \phi_{j}-\phi_{i}-{\tilde{\phi }}_{ij} \end{array}\right]$$

If the (i)th landmark is matched with the (j)th landmark, the relative pose measurement ${\tilde{T}}_{ij}$ is:

(16) $${\tilde{T}}_{ij}=I_{4\times 4}$$

Then, the pose error is:

(17) $${\tilde{T}}_{ij}^{-1}T_{ij}=\left[\begin{array}{cc} R\left(\phi_{j}-\phi_{i}\right) & R^{T}\left(\phi_{i}\right)\left(t_{j}-t_{i}\right)\\ 0 & 1 \end{array}\right]$$

The cost function in terms of position and angle for the matched landmarks $e_{ij}^{M}$ is defined as:

(18) $$e_{ij}^{M}=\left[\begin{array}{c} R^{T}\left(\phi_{i}\right)\left(t_{j}-t_{i}\right)\\ \phi_{j}-\phi_{i} \end{array}\right]$$

Therefore, the total error estimation function f is defined as follows [40]:

(19) $$f=\underset{\left[i,j\right]\in \Phi_{P}^{P}}{\sum }e_{ij}^{A}{}^{T}w_{P}^{P}e_{ij}^{A}+\underset{\left[i,j\right]\in \Phi_{P}^{S}}{\sum }e_{ij}^{A}{}^{T}w_{P}^{S}e_{ij}^{A}+\underset{\left[i,j\right]\in \Phi_{P}^{W}}{\sum }e_{ij}^{A}{}^{T}w_{P}^{W}e_{ij}^{A}+\underset{\left[i,j\right]\in \Phi_{S}^{S}}{\sum }e_{ij}^{M}{}^{T}w_{S}^{S}e_{ij}^{M}+\underset{\left[i,j\right]\in \Phi_{W}^{W}}{\sum }e_{ij}^{M}{}^{T}w_{W}^{W}e_{ij}^{M}$$

The importance of the association and matching constraints on map optimization is reflected by the information matrices $w_{P}^{P}$, $w_{P}^{S}$, $w_{P}^{W}$, $w_{S}^{S}$, and $w_{W}^{W}$, respectively. Particularly, the matching constraints’ information matrix satisfies the following constraint:

(20) $$0\leq w_{W}^{W}\leq w_{S}^{S}\leq 1$$

The graph optimization aims at finding a $\beta_{f}=\left[\begin{array}{c} t_{f}\\ \phi_{f} \end{array}\right]$ that minimize the error function f:

(21) $$\beta^{*}=argmin\left(f\right)$$

Then, a Gauss–Newton algorithm [51] is used here to solve the optimization problem, as presented in Equation (22):

(22) $$\beta^{k+1}=\beta^{k}-H^{-1}\nabla f$$

where H is a Hesse matrix of function f and ∇f is the value of function f’s gradient vector at point $\beta_{f}$.

Similar to the map alignment, the graph optimization is performed incrementally.

### 3.5. Venue Map Updating

In the overlapped areas of the candidate and venue maps, the optimized results are duplication. Additionally, the venue map lacks the context that is exclusive to the candidate map. Once a new iteration of graph optimization is complete, the venue map is updated in terms of the waypoints, semantic landmarks, Wi-Fi landmarks, and the association relationship between them.

The waypoints on a candidate map that matches the venue map adhere to the Euclidean distance principle [2] and heading principle, as shown in Equations (23) and (24):

(23) $$\sqrt{{\left(x_{a}^{V}-x_{b}^{C}\right)}^{2}+{\left(y_{a}^{V}-y_{b}^{C}\right)}^{2}}-d_{\varepsilon }\leq 0$$

(24) $$h_{a}^{V}-h_{b}^{C}-h_{\varepsilon }\leq 0$$

where $\left(x_{a}^{V},y_{a}^{V}\right)$ represents the position of a waypoint/landmark in a venue map and $\left(x_{b}^{C},y_{b}^{C}\right)$ denotes the position of a waypoint/landmark in a candidate map. $d_{\varepsilon }$ denotes the threshold of the Euclidean distance and $h_{\varepsilon }$ denotes the heading threshold of a camera. Once a waypoint in a candidate map satisfies Equations (23) and (24), the maturity of the corresponding waypoint on the venue map, $m^{V}$, is incremented by one, and its location is updated according to Equation (25). Otherwise, the unmatched waypoints from the candidate map are added directly to the venue map without modification to their maturity or location.

(25) $$\left(x_{a}^{V},y_{a}^{V}\right)=\left[\lambda_{V}\left(x_{a}^{V},y_{a}^{V}\right)+\lambda_{C}\left(x_{b}^{C},y_{b}^{C}\right)\right]/\left(\lambda_{V}+\lambda_{C}\right)$$

In Equation (25), $\lambda_{V}$ and $\lambda_{C}$ denote the weight factor of the venue and candidate maps, respectively, which are determined by the maturity of waypoints.

**Figure 5:**
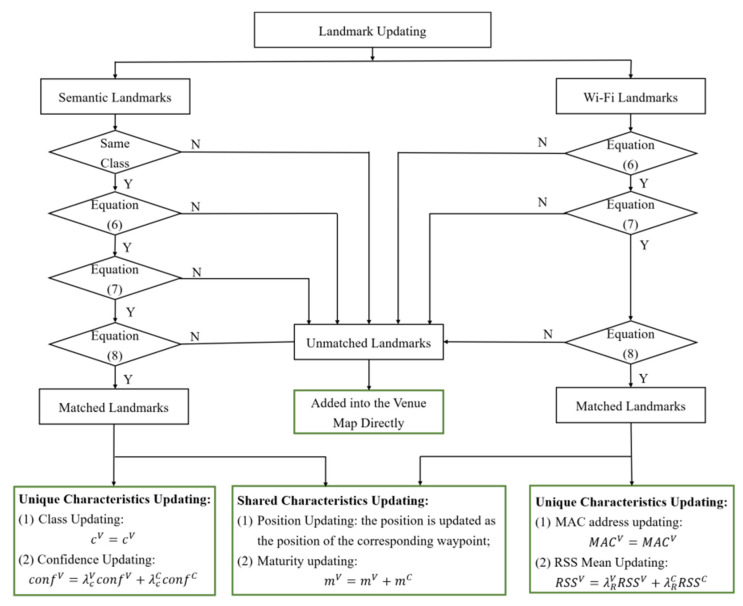
The flow chart of landmark updating.

The landmarks on a venue map are updated, as shown in Figure 5. Similarly, unmatched landmarks from a candidate map are added directly to the venue map with a lower maturity level than landmarks from the previous venue map. Therefore, these landmarks carry less weight in landmark matching-based map construction and localization. The unique and shared characteristics of landmarks that are matched on a venue map are updated. The class of a matched semantic landmark in a venue map $c^{V}$ remains unchanged. While its corresponding confidence $conf^{V}$ is calculated by weighted averaging the original confidence of the venue map $conf^{V}$ and the candidate map $conf^{C}$. The weight factor of the venue and candidate maps are $\lambda_{c}^{V}$ and $\lambda_{c}^{C}$, respectively. Similarly, the RSS mean value of a Wi-Fi landmark on a venue map is updated by weighted averaging the RSS value of the venue map $RSS^{V}$ and the candidate map $RSS^{C}$. The position of a landmark is updated as the location of the associated waypoint. Particularly, the maturity of a landmark in a venue map $m^{V}$ is continuously increased as it is matched with a landmark in a candidate map.

Finally, after updating all of the waypoints and landmarks, the association relationship between the waypoints, semantic landmarks, and Wi-Fi landmarks is updated.

### 3.6. Localization

Additionally, the updated venue map is utilized for localization. Similar to the preprocessing of crowdsourcing data, a local map is constructed by establishing an association between waypoints, semantic landmarks, and Wi-Fi landmarks. The transformation matrix $T_{L}^{V}$ between the local and venue maps is estimated in real-time using landmark matching. After transformation, a pedestrian’s location at current time t on the venue map $Pos_{t}^{V}$ is calculated as follows [18]:

(26) $$Pos_{t}^{V}=T_{L}^{V}Pos_{t}^{L}$$

where $Pos_{t}^{L}$ denotes a pedestrian’s location on a local map.

## 4. Experiment and Result

### 4.1. Experiment Setup

**Figure 6:**
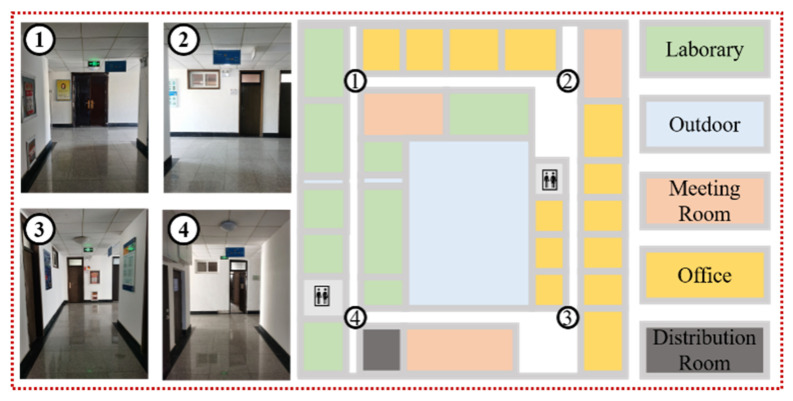
Schematic diagram of the office building scene.

**Figure 7:**
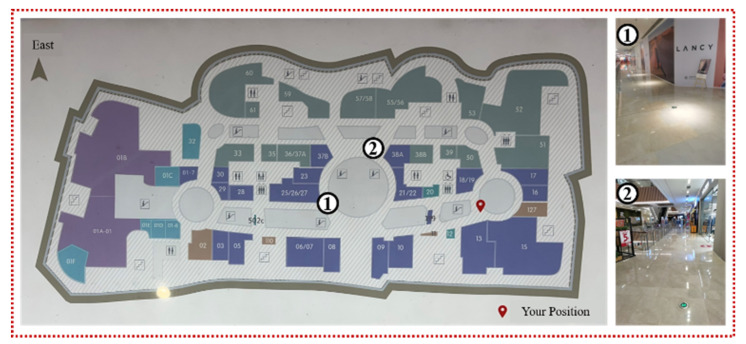
Schematic diagram of the shopping mall.

A series of experiments are conducted in a Qingdao office building (Figure 6) and a shopping mall (Figure 7) to evaluate the performance of the crowdsourcing-based indoor semantic map construction and localization method. We only collect experimental data from the fourth floor of the office building and a portion of the second floor of the shopping mall because it is time-consuming to manually collect crowdsourcing data for the entire office building and shopping mall scenes. The size of the shopping mall scene is nearly 700.0 m × 325.0 m (length × width), which is much larger than that of the office building scene. The length and width of the office building scene are nearly 70.0 m and 56.0 m, respectively. Four participants carrying a smartphone walked normally along predetermined experimental routes to collect sensor data via crowdsourcing, including images, IMU measurements, and Wi-Fi fingerprints. The diversity of experimental data is increased due to the participants’ varied walking habits, step lengths, and speeds.

We developed a client application to collect crowdsourcing data for experimental scenes that were predetermined. The first 90% of the collected data is preprocessed in the cloud, which was used for map construction via crowdsourcing. Localization relies on the remaining data collected by participants along the same route. Additionally, in order to obtain the ground truth for experimental validation, we recorded an additional reference video, which was captured by an assisting person who walked within a short distance from the actual collector. Before collecting the experimental data, a ground coordinate system was established using fixed-size tiles on the ground. The reference video and the pre-defined ground coordinate system allowed us to determine the relative location of the actual collector when his/her feet hit the ground.

### 4.2. Performance Evaluation of Crowdsourcing-Based Map Construction

**Figure 8:**
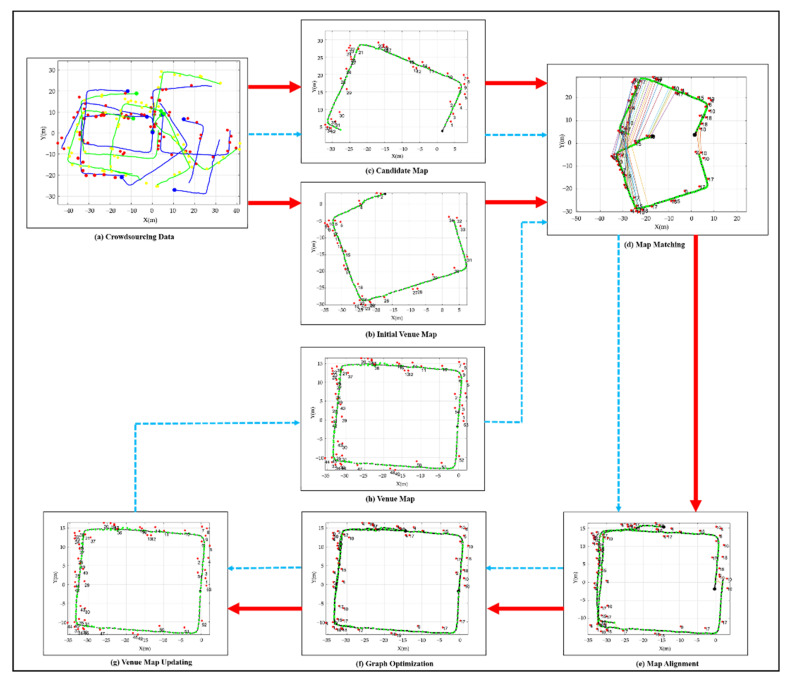
The process of venue map construction. The black circles denote the waypoints, red circles denote the semantic landmarks, and green circles denote the Wi-Fi landmarks.

Office building and shopping mall scenes were used to validate the proposed semantic map construction method. Figure 8 depicts the process of constructing and updating a venue map, using an office building as an example scene. Figure 8a depicts the single-track semantic maps obtained by preprocessing the crowdsourcing data. All the single-track semantic maps are evaluated by a map quality evaluation function and sorted according to their corresponding scores. Then, the single-track semantic map with the highest quality is chosen as the initial venue map, as presented in Figure 8b. Using the matched semantic and Wi-Fi landmarks between the remaining single-track semantic maps and the initial venue map, the map matching quality is evaluated. Then, the single-track semantic map with the highest matching quality with the venue map is chosen as the candidate map, as presented in Figure 8c. In Figure 8d, the matched semantic landmarks between the candidate and venue maps are connected with colored straight lines. The maps aligned after transformation are presented in Figure 8e. In Figure 8f, graph optimization further enhanced the fit of maps. After optimization, the venue map was updated, as presented in Figure 8g. The updated venue map, as presented in Figure 8h, is incorporated into the loop for the subsequent fusion and update. It is notable that, the red line denotes the initial selection and update of the venue map, and the blue line denotes the subsequent fusion and update of the venue map after the first iteration.

**Figure 9:**
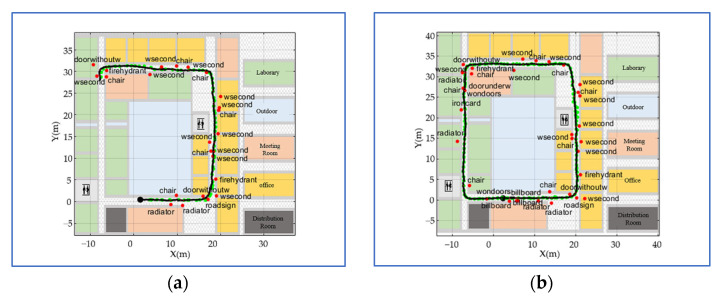
The comparison between the initial and fused venue maps in the office building scene. (**a**) The initial venue map, and (**b**) the fused venue map.

Compared to the initial venue map presented in Figure 9a, the fused venue map in Figure 9b covers the entire fourth floor of the office building. The number of semantic landmarks also increases from 24 to 35 after continuous fusion. As presented in Figure 9a,b, semantic landmarks are non-uniformly scattered on the venue map, which is consistent with the distribution of semantics on real venues. However, the distribution density of semantic landmarks on the venue map is lower than that on real venues. There are two reasons: (1) the training dataset cannot contain the semantic objects of all classes; (2) limited by the accuracy of the object detection methods, many small-sized semantic objects, such as exits, cannot be detected accurately and continuously. The trajectory after continuous fusion is much smoother than the initial trajectory; this is because the initial trajectory estimated by the PDR-aided VI-SLAM may be affected by scale or attitude drifts.

**Figure 10:**
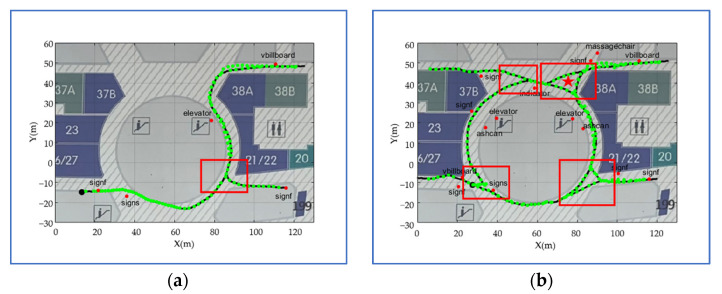
The comparison between the initial and fused venue maps in the shopping mall scene. (**a**) The initial venue map, and (**b**) the fused venue map.

The progress of constructing a venue map in the shopping mall scene is similar to that in the office building scene. After fusing all the crowdsourcing data, the fused venue map is presented in Figure 10b. Compared to the office building scene, the semantic density in the shopping mall scene is much sparser for there are few identifiable semantic objects. Particularly, the number of semantic landmarks increases from 5 to 15, and the semantic landmarks are scattered on the venue map. The most notable feature of the shopping mall scene is that there are lots of forks in the scene, which are marked with red boxes. The trajectories containing different forks are accurately and smoothly spliced while iterating. The spliced trajectory in Figure 10b reflects walkable routes and walking habits of pedestrians, such as dodging pillars (red pentagram).

### 4.3. Performance Evaluation of Localization

The localization error at the current time t $err_{t}$ is determined by the ground truth $GT_{t}$ and the positioning result corresponding to the ground truth timestamp $Pos_{t}$, as defined in Equation (27):

(27) $$err_{t}=GT_{t}-Pos_{t}$$

**Figure 11:**
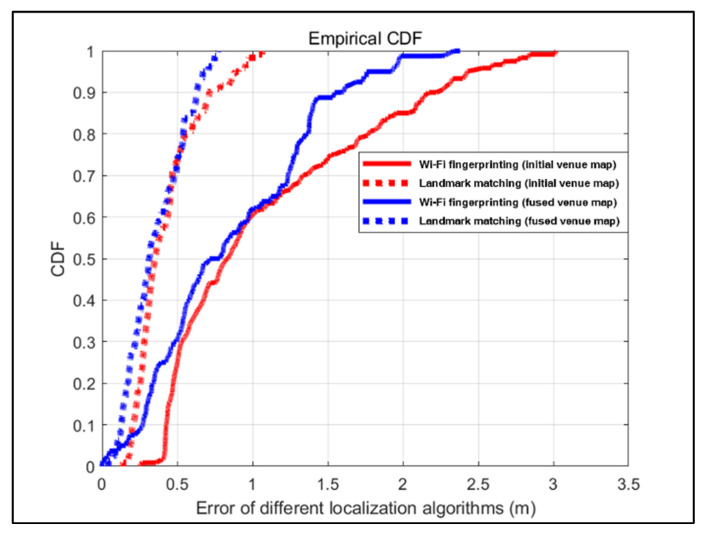
CDF of the location estimation error for the office building scene.

**Figure 12:**
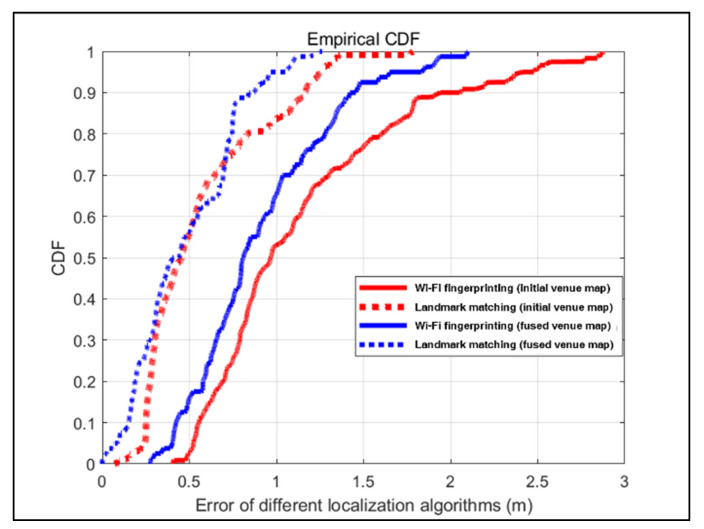
CDF of the location estimation error for the shopping mall scene.

In this paper, we calculate the location error cumulative distribution function (CDF) to evaluate the efficiency of the two localization methods, Wi-Fi fingerprinting-based localization, and landmark matching-based localization. Additionally, the initial and merged venue maps are utilized as localization maps, respectively. Figure 11 and Figure 12 illustrate the localization error for Wi-Fi fingerprinting-based localization in the initial venue map, landmark matching-based localization in the initial venue map, Wi-Fi fingerprinting-based localization in the fused venue map, and landmark matching-based localization in the fused venue map.

Compared to using the initial venue map as the localization map for the office building scene, the localization methods have higher localization accuracy in the fused venue map, as shown in Figure 11. Particularity, the Wi-Fi fingerprinting-based localization has an average localization error of 1.08 m with a standard deviation of σ=0.69 m in the initial venue map, while its localization error in the fused venue map is 0.87 m, and the standard deviation is σ=0.53 m. Compared to the initial venue map, the fused venue map’s Wi-Fi fingerprinting-based localization is 19.4% more accurate in terms of localization. Also compared are the localization results of the landmark matching-based localization, whose localization accuracy in the fused venue map is enhanced by over 14.2% compared to that in the initial venue map. Figure 11 demonstrates that landmark matching-based localization is more accurate than Wi-Fi fingerprinting-based localization. In the initial venue map, 90% of the CDF error for Wi-Fi fingerprinting-based localization is within approximately 2.2 m, while it is within approximately 0.7 m for landmark matching-based localization. In the fused venue map, 90% of the CDF error for Wi-Fi fingerprinting-based localization is within approximately 1.55 m, while it is within approximately 0.6 m for landmark matching-based localization.

For the shopping mall scene, compared to using the initial venue map as the localization map, the localization methods also have higher localization accuracy in the fused venue map, as shown in Figure 12. Particularity, the Wi-Fi fingerprinting-based localization has an average localization error of 1.15 m with a standard deviation of σ=0.57 m in the initial venue map, while its localization error in the fused venue map is 0.91 m. Compared to the initial venue map, the localization accuracy of the fused venue map’s Wi-Fi fingerprinting-based localization is improved by more than 20.8%. The localization results of the landmark matching-based localization are also compared, revealing that its localization accuracy in the fused venue map is enhanced by more than 16.0% compared to that in the initial venue map. Similarly, in the shopping mall scene, landmark matching-based localization is more accurate than Wi-Fi fingerprinting-based localization. In the initial venue map, 90% of the CDF error for Wi-Fi fingerprinting-based localization is within approximately 2.0 m, while it is within approximately 1.2 m for landmark matching-based localization. In the fused venue map, 90% of the CDF error for Wi-Fi fingerprinting-based localization is within approximately 1.45 m, while it is within approximately 0.85 m for landmark matching-based localization.

The average localization error comparison of the office building and shopping mall scenes is summarized in Table 2. It can be conducted that, compared to the office building scene, the proposed localization methods have higher average localization error in the shopping mall scene, especially for the landmark matching-based localization. Particularly, using the initial venue map as the localization map, the landmark matching-based localization has an average localization error of 0.42 m in the office building scene, while its average localization error in the shopping scene is 0.56 m. The localization error is increased by 0.14 m. Using the fused venue map as the localization map, the average localization of the landmark matching-based localization in the shopping mall scene is 0.11 m larger than that in the office building scene. However, the error between the average localization error of the Wi-Fi fingerprinting-based localization in the office building and shopping mall scenes is less than 0.1 m. The reason is that the semantic density of the shopping mall scene is much lower than that of the office building scene. Additionally, since the relative location of Wi-Fi landmarks is equal to the location of waypoints with the closest timestamp to the Wi-Fi landmarks, it may lead to a far distance between the waypoints matching the same Wi-Fi landmark in an empty shopping mall scene and increase the localization error.

## 5. Conclusions and Discussions

This paper proposes a crowdsourcing-based method, which solves the problem of indoor map construction and localization for unknown environments. The method utilizes the smartphones’ built-in sensors, such as cameras, IMUs, and Wi-Fi, so the system’s hardware cost is not increased. Smartphone-collected crowdsourcing data is preprocessed to construct single-track semantic maps. Those maps are evaluated using the proposed map quality evaluation function, and the highest quality one is chosen as the initial venue map. A candidate map having the highest matching degree with the selected venue map is selected for map fusion. After map alignment, the candidate and venue maps are optimized while satisfying the constraints. Inspired by the construction of single-track semantic maps, the optimization graph is constructed using waypoints, semantic landmarks, and Wi-Fi landmarks as nodes and the relevance between waypoints and landmarks as constraints. The venue map is lightweight since it is updated with respect to waypoints, semantic landmarks, Wi-Fi landmarks, and the association between them. In this paper, the construction and update of the venue map are performed using an incremental, iterative approach. A series of experiments are conducted in office building and shopping mall scenes. The results indicate that the venue map constructed using crowdsourcing data covers nearly all passed areas and can filter out incorrectly identified semantics to improve the map accuracy. The constructed venue map can also be used for multi-scene localization, with an average localization error of less than 0.5 m in the office building scene and 1.0 m in the shopping mall scene.

Since the proposed method is based on crowdsourcing, it has a high demand for sensor data. Collecting crowdsourcing data with a single smartphone platform is time-consuming and labor-intensive. Therefore, in the future, we will try multi-platform collaborative mapping methods, such as smartphones, robots, and UAVs. Low-quality single-track semantic maps can significantly reduce the venue map accuracy. Therefore, we will propose an efficient semantic map pre-screening mechanism before map fusion. The proposed optimization graph can be improved by using the constraints between semantic landmarks in a single-track semantic map as an added edge. At the same time, the constraint edge between semantic landmarks can also be utilized for landmark matching-based localization.

## Figures and Tables

**Table 1:** The attributes of semantic objects and feature points.

Attribute	UTC Time	2D Pixel Coordinate	3D Space Coordinate	Confidence	Class
Semantic Object	√	√	×	√	√
Feature Point	√	√	√	×	×

**Table 2:** The average localization error comparison of the office building and shopping mall scenes.

Method	Office Building (m)	Shopping Mall (m)
Wi-Fi fingerprinting (initial venue map)	1.08	1.15
Landmark matching (initial venue map)	0.42	0.56
Wi-Fi fingerprinting (fused venue map)	0.87	0.91
Landmark matching (fused venue map)	0.36	0.47

## Data Availability

Not applicable.
